# Predicting clinical outcomes after total knee arthroplasty from preoperative radiographic factors of the knee osteoarthritis

**DOI:** 10.1186/s12891-019-3029-7

**Published:** 2020-01-06

**Authors:** Kaoru Toguchi, Arata Nakajima, Yorikazu Akatsu, Masato Sonobe, Manabu Yamada, Hiroshi Takahashi, Junya Saito, Yasuchika Aoki, Toru Suguro, Koichi Nakagawa

**Affiliations:** 1 Department of Orthopaedic Surgery, Toho University Sakura Medical Center, 564-1 Shimoshizu, Sakura-city, Chiba, 285-8741 Japan; 2 Department of Orthopaedic Surgery, Sawara Prefectural Hospital, I-2285 Sawara, Katori-city, Chiba, 287-0003 Japan; 3 Department of Orthopaedic Surgery, Eastern Chiba Medical Center, 3-6-2 Okayamadai, Togane-city, Chiba, 283-8686 Japan; 4 Japan Research Institute of Artificial Joint, 725-1 Sugo, Kisarazu-city, Chiba, 292-0036 Japan

**Keywords:** Prediction, Patient-reported outcomes (PROs), Total knee arthroplasty (TKA), Radiograph, Osteoarthritis

## Abstract

**Background:**

Total knee arthroplasty (TKA) is the major surgical treatment for end-stage osteoarthritis (OA). Despite its effectiveness, there are about 20% of patients who are dissatisfied with the outcome. Predicting the surgical outcome preoperatively could be beneficial in order to guide clinical decisions.

**Methods:**

One-hundred and ten knees of 110 consecutive patients who underwent TKAs for varus knees resulting from OA were included in this study. Preoperative varus deformities were evaluated by femorotibial angle (FTA), medial proximal tibial angle (MPTA) and lateral distal femoral angle (LDFA), and classified as a severe varus (SV) or a mild varus (MV) group. The osteophyte score (OS), which we developed originally, was also calculated based on the size of the osteophytes and classified as groups with more or less osteophytes. We compared preoperative and 1-year postoperative range of motion, the Knee Society Score, and Japanese Knee injury Osteoarthritis Outcome Score (KOOS) between SV and MV groups (varus defined by FTA, MPTA, or LDFA), in each group with more or less osteophytes.

**Results:**

When varus deformities were defined by FTA, regardless of OS, postoperative KOOS subscales and/or the improvement rates were significantly higher in the SV group than in the MV group. When varus defined by MPTA, regardless of OS, there were no significant differences in postoperative KOOS subscales between groups. However, when varus defined by LDFA, scores for pain, activities of daily living (ADL), and quality of life (QOL) on postoperative KOOS and/or the improvement rates were significantly higher in the SV group than in the MV group only in patients with less osteophytes. No significant differences were found between groups in patients with more osteophytes.

**Conclusions:**

We classified OA types by radiographic measurements of femur and tibia in combination with OS. Postoperative patient-reported outcomes were better in patients with SV knees but were poor in patients with knees with MV deformity and less osteophytes.

## Background

Total knee arthroplasty (TKA) is the major surgical treatment for end-stage osteoarthritis. The procedure relieves patients of pain and improves their quality of life. Despite good clinical evaluation by physicians, it has been reported that approximately 20% of patients are dissatisfied with the outcome [1]. Thus, there is sometimes a dissociation between physician-based and patient-based outcomes in TKAs. There is great merit in being able to preoperatively identify patients who would have satisfactory or unsatisfactory results. Predicting the surgical outcomes preoperatively could be beneficial to guide clinical decisions.

Many studies have assessed the influence of surgical procedures or implant differences on outcomes after TKAs [2–9]. These factors vary depending on the surgeons’ experiences or the equipment used. Preoperative factors, however, do not vary by experience or surgical technique, so that they may directly predict the postoperative outcomes. Mental and emotional health influence postoperative patient-reported outcomes (PROs) [10, 11]. Also, preoperative pain and functional status, as measured by PROs, have been shown to predict pain and functional ability after TKAs [12, 13]. There are some reports focused on radiological factors of the knee, such as bone morphology, knee alignment and osteophytes, but they evaluate only the variation of radiographic factors themselves or the relationships between the factors and the progression of osteoarthritis [14–18]. Thus far, there is little information regarding the influence of preoperative radiological differences on PROs after TKA.

In this study, we classified patients with varus knee deformities in combination with preoperative radiographic factors and discuss what radiographic characteristics would predict satisfied or dissatisfied patients after TKAs.

## Methods

### Patients

A total of 110 consecutive patients (19 males and 91 females) who underwent primary total knee arthroplasties (TKAs) (110 knees) for varus knees resulting from osteoarthritis (OA) at our institution between January 2015 and December 2016 were included in this study. The exclusion criteria included valgus deformity, occurrence of fractures in lower limbs receiving TKAs, or progression of dementia during the follow-up period. Preoperative patient demographics and knee physical function indicators such as deformities, range of motion (ROM), and Knee Society Score (KSS) are shown in Table 1.

**Table 1:** Patients’ demographics, preoperative deformities, ROM, KSS, and KOOS

Number of patients (male/female)	110 (19/91)
Age, years old	73.0 ± 8.0
BMI, kg/m^2^	27.3 ± 4.3
Follow-up period, months (range)	12.1 (11–16)
FTA, degrees	185.1 ± 5.1
MPTA, degrees	83.9 ± 3.2
LDFA, degrees	81.0 ± 2.1
OS	6.5 ± 1.8
Extension, degrees	−10.5 ± 10.5
Flexion, degrees	120.5 ± 14.2
ROM, degrees	109.6 ± 21.4
KSS-KS	45.5 ± 14.7
KSS-FS	41.6 ± 18.3
KOOS-S	46.8 ± 18.8
KOOS-P	41.3 ± 18.2
KOOS-A	59.7 ± 17.3
KOOS-Q	26.3 ± 14.9

*FTA* femorotibial angle, *MPTA* Medial proximal tibial angle, *LDFA* Lateral distal femoral angle, *OS* Osteophyte score, *RO*M Range of motion; *KSS* Knee Society Score, *KS*, Knee score, *FS* Function score, *KOOS* Knee injury and Osteoarthritis Outcome Score. Values are expressed as mean ± SD

### Surgical procedures

All implants used in this study were the cruciate-retaining type FINE total knee (Teijin-Nakashima Medical, Okayama, Japan). Surgeries were performed according to our procedures published previously [19]. Briefly, osteophytes in the femorotibial and the patellofemoral joint were removed prior to cutting the distal femur and the proximal tibia, then distal femoral osteotomy was conducted perpendicular to the mechanical axis, and the posterior condyle was osteotomized parallel to the surgical epicondylar axis; a tibial osteotomy was subsequently conducted perpendicular to the anatomical axis of the tibia. Following osteotomy, adjustments for soft tissue balancing were performed before the implants were fixed to the bone with cement. Finally, the excess bone around femoral, tibial, and patellar implants was trimmed.

### Radiographic examinations and classification of patients

**Fig. 1:**
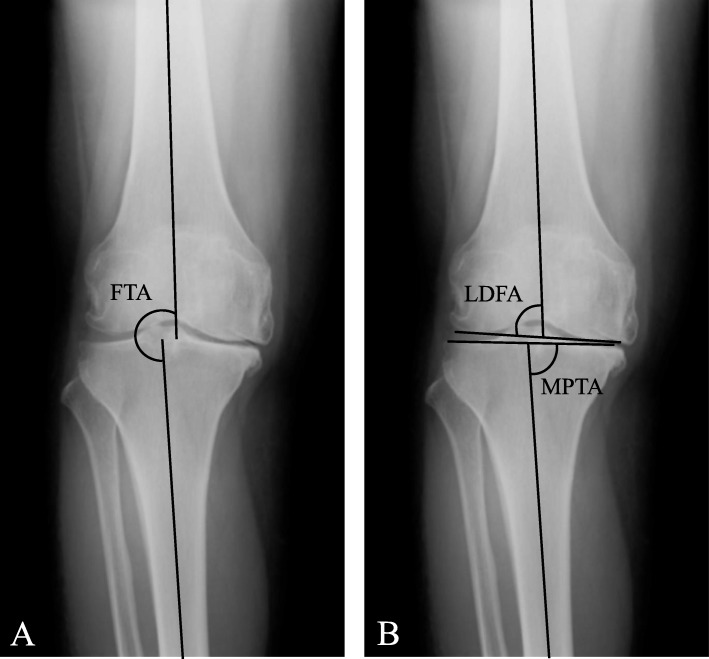
Radiographic measurement of femorotibial angle (FTA, **a**), medial proximal tibial angle (MPTA, **b**), and lateral distal femoral angle (LDFA, **b**) on an anteroposterior x-ray view of the knee

We measured preoperative femorotibial angle (FTA, Fig. 1a), medial proximal tibial angle (MPTA, Fig. 1b), and lateral distal femoral angle (LDFA, Fig. 1b) on standing anteroposterior x-ray views of the knee for all patients. When varus deformities were defined by FTA, patients with FTA≧185 ^o^ and FTA < 185 ^o^ were classified as a severe varus (SV) or a mild varus (MV) group, respectively since the average of FTA for all patients was 185.1 ± 5.1 degrees (Table 1). When varus was defined by MPTA or LDFA, the reference angle was determined based on the report of Nakano et al. [17]. When varus was defined by MPTA, patients with MPTA< 85 ^o^ and MPTA≧85 ^o^ were classified into the SV or the MV group, respectively. When varus was defined by LDFA, patients with LDFA≧82 ^o^ and LDFA< 82 ^o^ were classified into the SV or the MV group, respectively.

We developed the original osteophyte score (OS) that was calculated based on the size of osteophytes in the medial femorotibial, lateral femorotibial, and patellofemoral joints. The size of osteophytes was measured using a SYNAPSE-PACS software (FUJIFILM, Tokyo, Japan). Osteophyte scoring was performed as follows: none: 0 points; < 3 mm: 1 point; 3–5 mm: 2 points; > 5 mm: 3 points. A total score ≧7 points or < 7 points was defined as groups with more osteophytes or less osteophytes, respectively since the average of the OS for all patients was 6.5 ± 1.8 (Table 1).

Patients were first classified into groups with more or less osteophytes, then further classified into SV or MV subgroups within each osteophyte group according to the definition of varus deformities; i.e., FTA, MPTA, or LDFA.

### Evaluation of clinical and patient-reported outcomes (PROs)

We used the KSS as an objective evaluation of clinical outcomes, which consists of the Knee Score (KS) and the Function Score (FS). In addition to the KSS, we examined the Japanese Knee injury Osteoarthritis Outcome Score (KOOS), an instrument of confirmed validity and reliability for patient-reported outcomes (PROs) based on its cross-cultural adaptation [20]. The KOOS consists of a total of 42 knee-related items. Each item was scored from 0 to 4. Five subscales including symptoms, pain, activities of daily living (ADL), sports/recreation, and quality of life (QOL) were converted to 100 points [21]. In this study, 4 subscales except for sports/recreation were evaluated preoperatively and 1-year postoperatively. The improvement rate was calculated as gain of points/ (100-preoperative points). This study was approved by the institutional review board at Toho University Sakura Medical Center (application number: S17012). All activities were performed in accordance with the ethical standards set forth in the Declaration of Helsinki.

### Statistical analyses

The reliability of each radiographic measurement was assessed using intraclass correlation coefficients. One resident (KT) and 2 consultants (AN and YoA) performed the measurements from 10 knees for inter-observer repeatability and from another 10 knees for intra-observer repeatability. All radiographic measurements in this study showed good reliability (all values > 0.8). Results were expressed as the mean ± standard deviation. Correlations between variables were analyzed using a Pearson’s correlation coefficient. We compared preoperative and postoperative ROM, the KSS, and KOOS subscales between SV and MV groups in each group with more or less osteophytes using a Mann-Whitney U test. Data analyses were performed using SPSS software, version 21 (SPSS Inc., Chicago, IL, USA) and *p*-values of < 0.05 were considered statistically significant.

## Results

### Correlations among the radiographic factors

A moderate correlation was found between FTA and MTPA (*r* = − 0.44) (Table 2). Weak correlations were found between FTA and LDFA (*r* = 0.37) and between FTA and OS (*r* = 0.28). No significant differences were found between MPTA and LDFA, between LDFA and OS, or between MPTA and OS.

**Table 2:** Correlations among the radiographic factors

	FTA	MPTA	LDFA	OS
FTA		−0.44*	0.37*	0.28*
MPTA			n.s.	n.s.
LDFA				n.s.
OS				

Pearson’s correlation coefficient. *Statistically significant, *p* < 0.05. n.s.: not significant, *FTA* Femorotibial angle, *MPTA*. Medial proximal tibial angle, *LDFA* Lateral distal femoral angle; *OS* Osteophyte score

### Comparison of the clinical outcome and PROs between the SV and the MV subgroups in groups with more or less osteophytes. Varus defined by FTA

In patients with more osteophytes, there were no significant differences in ROM, KS, and FS between the SV and the MV groups (Table 3). In patients with less osteophytes, the improvement rate of KS was significantly higher in the SV group than in the MV group.

**Table 3:** Comparison of clinical and patient-reported outcomes between SV and MV subgroups in more and less osteophytes groups. Varus defined by FTA

		More osteophytes (*n* = 47)			Less osteophytes (*n* = 63)		
		SV (*n* = 23)	MV (*n* = 24)	p-value	SV (*n* = 16)	MV (*n* = 47)	p-value
ROM	preoperative (degree)	97.0 ± 22.9	107.3 ± 20.0	0.11	114.1 ± 20.2	115.5 ± 19.5	0.8
	postoperative (degree)	120.7 ± 15.8	122.5 ± 10.9	0.65	123.4 ± 11.1	122.0 ± 12.9	0.67
	angle difference (degree)	23.7 ± 20.1	14.8 ± 15.9	0.1	9.4 ± 13.9	6.0 ± 20.1	0.46
KS	preoperative	37.4 ± 15.4	43 ± 12.0	0.18	43.5 ± 13.6	51.4 ± 13.9	0.08
	postoperative	96.3 ± 6.0	97.0 ± 4.1	0.61	97.7 ± 3.2	96.2 ± 5.2	0.19
	improvement rate (%)	94.3 ± 9.6	94.9 ± 6.66	0.81	95.7 ± 4.77	87.9 ± 22.0	0.03*
FS	preoperative	39.1 ± 18.1	40.9 ± 18.1	0.74	40.4 ± 14.8	43.7 ± 19.8	0.52
	postoperative	79.6 ± 13.2	73.3 ± 22.3	0.25	79.4 ± 16.2	76.2 ± 17.7	0.49
	improvement rate (%)	67.8 ± 19.9	58.1 ± 28.0	0.19	70.7 ± 24.1	53.9 ± 33.5	0.05
KOOS-	preoperative	42.5 ± 22.0	47.4 ± 18.7	0.42	46.6 ± 21.2	48.6 ± 16.6	0.75
Symptom	postoperative	83.8 ± 14.7	78.1 ± 15.4	0.21	83.8 ± 11.6	79.0 ± 17.0	0.23
	improvement rate (%)	72.3 ± 21.4	57.1 ± 32.6	0.07	67.7 ± 19.2	53.9 ± 36.6	0.07
KOOS-	preoperative	42.4 ± 19.3	43.4 ± 18.8	0.85	42.7 ± 18.1	39.1 ± 17.8	0.52
Pain	postoperative	89.2 ± 12.0	85.8 ± 15.7	0.41	92.3 ± 7.2	83.8 ± 14.0	0.003*
	improvement rate (%)	81.3 ± 18.2	76.6 ± 27.7	0.51	84.7 ± 14.7	70.3 ± 25.6	0.01*
KOOS-	preoperative	60.3 ± 14.6	59.3 ± 18.6	0.84	61.3 ± 13.2	59.2 ± 19.2	0.64
ADL	postoperative	89.8 ± 9.9	81.9 ± 16.3	0.06	90.4 ± 7.1	84.8 ± 12.2	0.03*
	improvement rate (%)	75.4 ± 22.0	54.3 ± 34.1	0.02*	75.5 ± 13.2	57.4 ± 34.9	0.005*
KOOS-	preoperative	24.9 ± 14.0	26.9 ± 17.0	0.67	21.4 ± 12.9	28.1 ± 14.9	0.11
QOL	postoperative	74.0 ± 20.3	58.4 ± 25.2	0.03*	67.4 ± 18.9	64.1 ± 21.0	0.56
	improvement rate (%)	66.4 ± 25.0	45.7 ± 28.1	0.01*	61.6 ± 20.8	48.1 ± 28.8	0.06

*SV* Severe varus. FTA > 185; *MV*: mild varus. FTA < 185; *FTA* Femorotibial angle, *ROM* Range of motion, *KS* Knee score, *FS* Function score, *KOOS* Knee injury and osteoarthritis outcome score. *Significantly different, p < 0.05

Additionally, in patients with more osteophytes, significantly higher QOL scores on the KOOS were obtained in the SV group than in the MV group. Furthermore, the improvement rates of scores for ADL and QOL on the KOOS were significantly higher in the SV group than in the MV group. In patients with less osteophytes, pain and ADL scores on the KOOS was significantly higher in the SV group than in the MV group. The improvement rates for scores of pain and ADL on the KOOS were significantly higher in the SV group than in the MV group.

### Comparison of the clinical outcome and PROs between SV and MV subgroups in groups with more or less osteophytes. Varus defined by MPTA

In patients with more osteophytes, the SV group had significantly higher ROM than the MV group (Table 4). There were no significant differences in KS or FS between the SV and the MV groups.

**Table 4:** Comparison of clinical and patient-reported outcomes between SV and MV subgroups in more and less osteophytes groups. Varus defined by MPTA

		More osteophytes (*n* = 47)			Less osteophytes (*n* = 63)		
		SV (*n* = 29)	MV (*n* = 18)	*p*-value	SV (*n* = 48)	MV (*n* = 15)	*p*-value
ROM	preoperative (degree)	105.0 ± 22.2	97.8 ± 21.2	0.27	113.8 ± 19.6	119.7 ± 19.1	0.31
	postoperative (degree)	125.7 ± 10.7	115.0 ± 15.0	0.01*	122.2 ± 11.5	123 ± 15.3	0.85
	angle difference (degree)	20.3 ± 19.4	17.2 ± 16.9	0.57	7.9 ± 19.6	3.3 ± 15.5	0.36
KS	preoperative	39.5 ± 13.4	41.5 ± 15.2	0.67	50.3 ± 15.0	47.5 ± 11.5	0.47
	postoperative	97.2 ± 5.6	95.8 ± 4.3	0.36	97 ± 4.3	95.4 ± 6.2	0.38
	improvement rate (%)	95.6 ± 8.9	93.0 ± 6.7	0.28	93.3 ± 9.9	79.2 ± 33.6	0.13
FS	preoperative	39.1 ± 18.7	41.6 ± 16.9	0.66	44.5 ± 17.5	37.7 ± 22.0	0.32
	postoperative	76.3 ± 15.6	76.7 ± 22.6	0.94	77.2 ± 16.9	75.7 ± 19.0	0.79
	improvement rate (%)	62.5 ± 23.3	63.7 ± 27.1	0.89	59.7 ± 32.6	51.9 ± 31.5	0.42
KOOS-	preoperative	42.6 ± 21.3	49.1 ± 18.6	0.29	50.1 ± 18.3	41.4 ± 13.8	0.06
Symptom	postoperative	80.3 ± 16.4	82.1 ± 13.3	0.68	81.9 ± 15.6	77.3 ± 16.9	0.44
	improvement rate (%)	66.8 ± 23.6	60.8 ± 35.5	0.54	56.7 ± 34.7	58.3 ± 31.8	0.87
KOOS-	preoperative	41.9 ± 18.3	44.6 ± 20.2	0.66	40.7 ± 17.3	37.7 ± 19.9	0.62
Pain	postoperative	86.1 ± 15.3	89.6 ± 11.6	0.39	86.1 ± 14.9	85.3 ± 14.9	0.85
	improvement rate (%)	77.1 ± 25.2	81.9 ± 20.3	0.49	74.7 ± 21.9	70.7 ± 30.6	0.65
KOOS-	preoperative	58.0 ± 14.0	62.6 ± 20.2	0.42	61.5 ± 20.5	53.7 ± 20.5	0.22
ADL	postoperative	85.6 ± 13.8	86.2 ± 14.6	0.89	85.9 ± 11.1	87.2 ± 12.2	0.72
	improvement rate (%)	68.2 ± 27.7	58.7 ± 34.6	0.34	60.5 ± 31.7	65.0 ± 34.1	0.65
KOOS-	preoperative	23.5 ± 14.0	29.8 ± 17.3	0.21	25.6 ± 14.2	29.4 ± 16.2	0.44
QOL	postoperative	64.0 ± 25.7	69.7 ± 21.1	0.42	66.6 ± 20.9	59.8 ± 18.1	0.24
	improvement rate (%)	55.2 ± 30.3	56.8 ± 25.7	0.85	54.9 ± 27.6	40.2 ± 25.2	0.07

SV: strong varus. MPTA< 85; MV: mild varus. MPTA> 85; MPTA: medial proximal tibial angle; ROM: range of motion; KS: knee score; FS: function score; KOOS: knee injury and osteoarthritis outcome score. *Significantly different, *p* < 0.05

In patients with less osteophytes, no significant differences in ROM, KS, or FS were found between the SV and the MV groups.

There were no significant postoperative differences in any of the KOOS subscales between the SV and the MV groups.

### Comparison of the clinical outcome and PROs between SV and MV subgroups in groups with more and less osteophytes. Varus defined by LDFA

In patients with more osteophytes, there were no significant differences in postoperative ROM, KS, or FS between the SV and the MV groups (Table 5). In patients with less osteophytes, the improvement rates of KS and FS were significantly higher in the SV group than in the MV group.

**Table 5:** Comparison of clinical and patient-reported outcomes between SV and MV subgroups in more and less osteophytes groups. Varus defined by LDFA

		More osteophytes (*n* = 47)			Less osteophytes (*n* = 63)		
		SV (*n* = 18)	MV (*n* = 29)	*p*-value	SV (*n* = 22)	MV 41)	*p*-value
ROM	preoperative (degree)	99.4 ± 20.9	104.0 ± 22.7	0.49	113.9 ± 20.0	115.9 ± 19.5	0.71
	postoperative (degree)	123.1 ± 13.6	120.7 ± 13.5	0.56	122.7 ± 12.8	122.2 ± 12.4	0.87
	angle difference (degree)	23.1 ± 19.3	16.8 ± 17.8	0.27	8.9 ± 16.1	5.7 ± 20.0	0.5
KS	preoperative	39.7 ± 11.9	40.5 ± 15.3	0.85	48.5 ± 13.9	50.3 ± 14.4	0.65
	postoperative	95.4 ± 6.8	97.5 ± 3.5	0.25	97.6 ± 3.4	96 ± 5.4	0.17
	improvement rate (%)	92.5 ± 10.8	96.0 ± 5.78	0.23	95.7 ± 5.3	86.3 ± 23.8	0.03*
FS	preoperative	31.8 ± 21.5	45.2 ± 13.2	0.03*	45.1 ± 19.3	41.6 ± 18.4	0.5
	postoperative	70.8 ± 23.3	80 ± 13.7	0.14	81.4 ± 18.1	74.3 ± 16.4	0.14
	improvement rate (%)	59.0 ± 26.3	65.5 ± 23.4	0.41	72.3 ± 26.1	49.4 ± 32.7	0.005*
KOOS-	preoperative	45.4 ± 18.7	44.8 ± 21.7	0.92	48.7 ± 21.8	47.7 ± 15.1	0.86
Symptom	postoperative	81.1 ± 15.4	80.9 ± 15.3	0.98	84.5 ± 11.2	77.9 ± 17.6	0.07
	improvement rate (%)	63.4 ± 28.9	65.3 ± 27.0	0.83	63.1 ± 31.2	53.9 ± 35.1	0.31
KOOS-	preoperative	45.4 ± 15.9	41.3 ± 20.7	0.45	40.5 ± 18.8	39.7 ± 17.5	0.89
Pain	postoperative	86.2 ± 17.4	88.4 ± 11.5	0.64	90.6 ± 8.2	83.3 ± 14.6	0.01*
	improvement rate (%)	76.9 ± 29.5	80.3 ± 18.7	0.67	81.8 ± 18.0	69.3 ± 26.0	0.03*
KOOS-	preoperative	59.8 ± 18.2	59.7 ± 15.7	0.98	63.9 ± 19.3	57.3 ± 16.9	0.2
ADL	postoperative	83 ± 18.4	87.7 ± 10.1	0.33	90.1 ± 8.3	84.1 ± 12.2	0.02*
	improvement rate (%)	59.4 ± 36.1	68.1 ± 26.2	0.39	72.2 ± 29.3	55.9 ± 32.4	0.06
KOOS-	preoperative	25.9 ± 19.5	25.9 ± 12.4	0.99	26.8 ± 15.2	26.4 ± 14.5	0.92
QOL	postoperative	66.5 ± 26.7	66 ± 22.6	0.95	74.9 ± 18.5	59.5 ± 19.4	0.004*
	improvement rate (%)	57.6 ± 29.0	54.6 ± 28.4	0.74	63.5 ± 28.9	44.6 ± 24.8	0.02*

*SV* Strong varus. LDFA> 82; *MV* Mild varus. LDFA< 82; *LDFA* Lateral distal femoral angle, *ROM* Range of motion, *KS* Knee score, *FS* Function score, *KOOS* Knee injury and osteoarthritis outcome score. *Significantly different, *p* < 0.05

Additionally, in patients with more osteophytes, there were no significant differences in any of the KOOS subscales between the SV and the MV groups. In patients with less osteophytes, postoperative pain, ADL, and QOL scores were significantly higher in the SV group than in the MV group. Furthermore, the improvement rates for scores of pain and QOL on the KOOS were significantly higher in the SV group than in the MV group.

## Discussion

In this study, correlations between FTA and OS, MPTA and OS, and between LDFA and OS were weak or not significant, suggesting that classification of patients by radiographic measurements of the femur and tibia in combination with OS provides additional information over the individual factors alone. Thus, we classified patients by FTA, MPTA, or LDFA in combination with OS. When varus deformities were defined by FTA, regardless of OS, postoperative KOOS subscales and/or the improvement rates were significantly higher in the SV group than in the MV group. When varus was defined by MPTA, there were no significant differences in postoperative KOOS subscales between groups; however, when varus was defined by LDFA, scores of pain, ADL, or QOL on the KOOS, and/or the improvement rates were significantly higher in the SV group than in the MV group only in patients with less osteophytes. Taken together, postoperative PROs are expected to be better in patients with SV knees.

Riis et al. showed vise versa: i.e., preoperative low-grade severity of OA was associated with a low functional level after TKA [22]. Based on this finding, they stated that avoiding premature surgery could assist in reducing the number of patients who are dissatisfied following TKA. Similar results have been shown by several investigators that less severe preoperative radiological OA was associated with a poorer outcome after TKAs [23–25].

Osteophytes affect ROM, pain, and function in patients with knee OA [26]. Therefore, we classified OA types by radiographic measurements in combination with OS. Higher preoperative KL grades were associated with better postoperative WOMAC scores [27]. Patients with more severe radiographic damage at the time of surgery are more likely to have substantial gains in terms of both pain relief and improved function as a result of a TKA [25]. These reports are consistent with the results obtained in this study. However, classification of OA types by varus severity in combination with OS has not been attempted to date. Sowers showed that large osteophytes, marked synovitis, macerated meniscal tears, and full-thickness tibial cartilage defects were associated with increased odds of knee pain and with 30–40% slower walking and stair-climbing times [26]. This suggests that osteophytes are associated with pain and physical functioning of knee OA patients. Therefore, we investigated whether the total size of osteophytes affected postoperative clinical results or patient-reported outcomes, and compared the postoperative KSS and KOOS subscales between groups with more or less osteophytes. Unexpectedly, there were no significant differences between these groups (data not shown). Then, we subdivided patients into the SV and the MV groups in each group with more or less osteophytes.

Previous publications have reported other factors than radiographic characteristics as factors predicting postoperative poor results. Lewis et al. reviewed 32 studies involving almost 30,000 patients and found that in addition to preoperative knee pain and pain at other sites, catastrophizing and mental health were the strongest independent predictors of persistent pain after TKA [28]. Khatib et al. reviewed 19 studies containing data on 9046 TKAs performed in 8704 adult patients and reported that the preoperative psychological state may affect the outcome after a TKA [29]. Furthermore, overweight [30], BMI [31, 32], age and preoperative KSS [33], anxiety and depression [34, 35] were important predictors for dissatisfaction after TKA. Taken together with the results obtained in this study, TKAs for patients with obesity or psychological disorders together with knees with MV deformity and less osteophytes should be avoided, or the patients should be referred for consultation to psychological experts before surgery.

This study has some limitations. First, several different surgeons (AN, YoA and KN) performed the TKAs, and surgical approaches varied among surgeons. Second, postoperative complications were not considered and psychological factors were not investigated. Third, statistical analyses were performed only between 2 groups and not among multi-groups. Nevertheless, classification of OA types by radiographic measurements of femur and tibia in combination with OS may allow surgeons to predict postoperative outcomes and to avoid TKAs with which patients would be dissatisfied.

In conclusion, we classified OA types by radiographic measurements of femur and tibia in combination with OS. Postoperative PROs were better in patients with knees with SV deformity but were poor in patients with knees with MV deformity and less osteophytes. Classification of knee OA types in this way may allow surgeons to select patients who would be satisfied or dissatisfied with TKAs. Further studies are required to elucidate in which OA types better or poor postoperative outcomes would be predicted from radiographic characteristics.

## Conclusions

We classified OA types by radiographic measurements of femur and tibia in combination with OS. Postoperative PROs were better in patients with SV knees but were poor in patients with knees with MV deformity and less osteophytes. Classification of knee OA types by the radiographic characteristics may allow surgeons to select patients who would be satisfied or dissatisfied with TKAs.

## Data Availability

All data generated or analyzed during the current. study are included in this published article.

## Abbreviations

FTA: Femorotibial angle
KOOS: Knee injury osteoarthritis outcome Score
KSS: Knee society score
LDFA: Lateral distal femoral angle
MTPA: Medial tibial proximal angle
MV: Mild varus
OA: Osteoarthritis
OS: Osteophyte score
PROs: Patient-reported outcomes
SV: Severe varus
TKA: Total knee arthroplasty
